# Biodegradation Study of Food Packaging Materials: Assessment of the Impact of the Use of Different Biopolymers and Soil Characteristics

**DOI:** 10.3390/polym16202940

**Published:** 2024-10-20

**Authors:** Amanda Martinello Neres de Souza, Luisa Bataglin Avila, Camila Ramão Contessa, Alaor Valério Filho, Gabriela Silveira de Rosa, Caroline Costa Moraes

**Affiliations:** 1 Graduate Program in Science and Engineering of Materials, Federal University of Pampa, 1650 Maria Anunciação Gomes de Godoy Avenue, Bagé 96413-172, Brazil; amandasouza.aluno@unipampa.edu.br (A.M.N.d.S.); gabrielarosa@unipampa.edu.br (G.S.d.R.); 2 Department of Chemical Engineering, Federal University of Santa Maria, Santa Maria 97105-900, Brazil; luisabataglinavila@gmail.com; 3 Chemical Engineering, Federal University of Pampa, 1650, Maria Anunciação Gomes de Godoy Avenue, Bagé 96413-172, Brazil; 4 Engineering and Science of Food Graduate Program, Laboratory Bioprocess Engineering, College of Chemistry and Food Engineering, Federal University of Rio Grande, Italy Avenue, km 08, Campus Carreiros, Rio Grande 96203-900, Brazil; camilaramao@hotmail.com; 5 Food Engineering, Federal University of Pampa, 1650, Maria Anunciação Gomes de Godoy Avenue, Bagé 96413-172, Brazil; 6 Graduate Program in Materials Science and Engineering, Technology Development Center, Federal University of Pelotas, 1 Gomes Carneiro, Pelotas 96010-610, Brazil; alaorvf@msn.com

**Keywords:** morphological analysis, natural polymer, soil microbiota

## Abstract

In this article, the relationship between the properties of different membranes (agar, chitosan, and agar + chitosan) and biodegradability in natural and sterilized soil was investigated. The membranes under investigation exhibited variations in the biodegradation process, a phenomenon closely linked to both the soil microbiota composition and their water affinity. Higher solubility in water and greater swelling tendencies correlated with shorter initiation times for the biodegradation process in soil. Overall, all tested membranes began biodegradation within 14 days, as assessed through thickness and morphological analysis parameters, demonstrating a superior degradation rate compared to low-density polyethylene films.

## 1. Introduction

The widespread use of synthetic plastics is constantly growing, leading to a serious problem of managing non-degradable solid waste. Studies show that plastic production has increased approximately 20 times in the last 50 years. Adding to this problem is the reality that a significant portion of the overall output remains untreated, worsening environmental pollution [1,2,3,4].

The increasing concern about the environmental damage caused by synthetic plastics, and their subsequent impact on human health, has prompted efforts to explore alternatives capable of mitigating these effects. In this context, the production of materials based on biopolymers has garnered attention, whether through the utilization of a single biopolymer or a combination of several [5,6,7].

Among the biopolymers, chitosan and agar-agar have been the focus for many researchers due to their favorable attributes and versatile applications. Chitosan, ranked as the second most abundant polymer after cellulose, exhibits properties such as antimicrobial and antioxidant effects, along with non-toxicity [8,9]. Additionally, agar-agar, a polysaccharide extracted from red algae, has characteristics such as biocompatibility and hydrophilicity [10,11].

Although biopolymers originate from renewable sources, not all of them yield biodegradable or compostable materials, i.e., bio-based plastics are different from biodegradable plastics. The biodegradability of plastics is linked to various factors, such as crystallinity and chemical structure. Biodegradation involves the breakdown of a polymer under environmental conditions, characterized by structural changes and deterioration of components. This process leads to the formation of other compounds, such as water, minerals, and carbon. In this process, some factors can influence the rate of degradation, such as thermal activation, hydrolysis, biological activity (i.e., enzymes), oxidation, photolysis, or radiolysis with the combined execution of biotic and nonbiotic processes. These factors directly affect the biopolymer degraded, and indirectly affect it through changes in the microbial population and the activity of the various microorganisms themselves due to humidity, temperature, pH, salinity, and presence or absence of oxygen. Besides that, the physical and chemical characteristics of biopolymeric material also contribute to the biodegradation process and include porosity, morphology, cross-linking, purity, chemical reactivity, mechanical strength, thermal tolerance, and resistance to electromagnetic radiation [12,13].

However, some authors erroneously label any material developed from biopolymers as biodegradable, despite limited studies evaluating this property using standardized methods [14,15,16]. Thus, this current research aimed to collaborate with studies on the biodegradability of bio-based films. From this, three bio-based films were produced using chitosan and agar-agar. Then, their biodegradability was evaluated using two different soils (natural and sterile).

## 2. Materials and Methods

### 2.1. Reagents

Chitosan (Oakwood Chemical, Estill, SC, USA), molar mass 170.7–198.5 kDa, degree of deacetylation 95% according to the manufacturer’s data, and agar-agar (Himedia, WF, Pelotas, RS, Brazil), glycerol (Alphatec, WF, Pelotas, RS, Brazil), and acetic acid (Synth, Diadema, SP, Brazil) were used to prepare the biopolymeric membranes. The soil samples used in the biodegradation analysis were supplied by Macsul Terraplanagem company, located at Bagé, Rio Grande do Sul, Brazil (−31.32850330385889, −54.09143894201339).

### 2.2. Preparation of Biopolymeric Membranes

Membranes were obtained using a casting technique with different proportions (Table 1) according to the method described by Contessa et al. [7], where the chitosan was dissolved in acetic acid (1 M) at room temperature and agar-agar was dissolved in distilled water at 80 °C. Then, the membranes were dried in a convective dryer set at 40 °C for 24 h. After production, the bio-based membranes were removed from the plates and kept at room temperature with 50% relative humidity.

### 2.3. Characterization of Membranes

**Figure 1:**
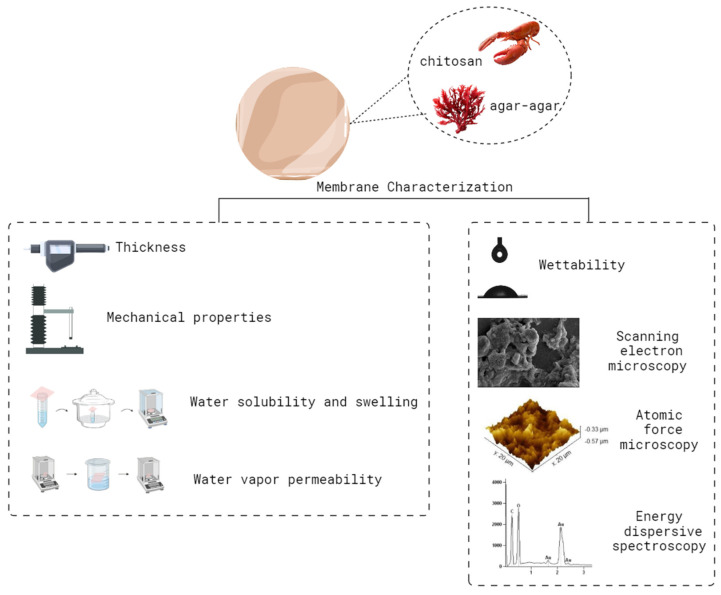
Summary of the analyses carried out to characterize the membranes.

Membranes were characterized according to the parameters described in Figure 1.

Thickness was measured using a digital micrometer (Insize-IP65, Boituva, SP, Brazil) at 10 random locations along the membranes and expressed as the average of these points. Following that, the membranes were evaluated for tensile strength (TS) and percentage of elongation at break (E) according to the American Society for Testing and Materials D 882-12 [17] using a texture analyzer (STABLE MICRO SYSTEM—TA.XT.plus, Surrey, UK). The membranes were also evaluated according to water solubility [18] and swelling [19], as well as water vapor permeability [20]. Determination of the hydrophilicity of the membranes was performed through contact angle analysis using an optical tensiometer (One Attension—Theta Instrument). Finally, the morphology was evaluated using scanning electron microscopy (SEM; Jeol, JSM-6610LVAkishima, Tokyo, Japan) at 500, 2500 and 5000× magnifications and atomic force microscopy (AFM; Agilent 5500, Agilent Technologies, Santa Clara, CA, USA) with Gwyddion 2.54 software for image acquisition. For analyses, the samples were fixed with double-sided carbon tape and placed in the sample holder. Chemical characterization was carried out by means of energy dispersive spectroscopy.

### 2.4. Biodegradation in Soil

**Figure 2:**
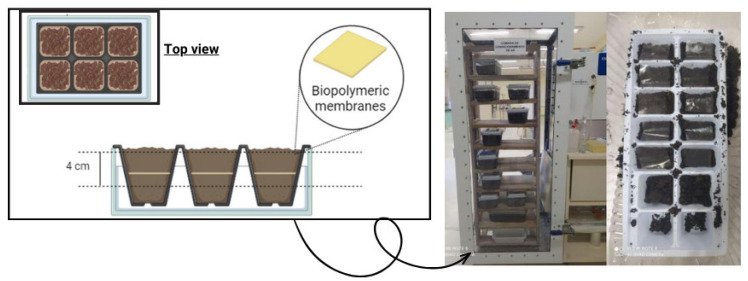
Experimental apparatus for analyzing biodegradation in soil.

The biodegradability of the membranes in soil was analyzed according to the method described by Martucci and Ruseckaite [21]. The samples were cut (2 × 3 cm) and placed into a container (441 cm^2^) containing soil samples at room temperature and were buried at 4 cm depth. The samples were conditioned inside a climate chamber (Ecoeducacional, São José, SC, Brazil), and every seven days water was sprayed to maintain soil moisture. Figure 2 shows the experimental apparatus for analyzing biodegradation in soil.

The tests were carried out in two ways: in soil collected naturally and slightly moistened, called “natural soil”, and in sterilized soil. This soil was obtained from the same region but underwent an autoclave sterilization process to eliminate the initial microbiota before the degradation test.

After the initial 7 days, a microbiological assessment was conducted (Section 2.5) to evaluate the microbiological profile of the soil composition, focusing on the simple counting of mesophilic aerobes, molds, and yeasts. The buried samples were evaluated for their visual appearance, by a comparison of images with the standard (the sample before being buried), and for reduction in thickness of the samples using a digital micrometer. This procedure was repeated for several days, totaling 112 days of the experiment.

### 2.5. Soil Characterization

Soil samples were evaluated for organic matter, moisture content, and microbial activity. The evaluation of organic matter and moisture content was obtained using soil particles with a diameter of less than 0.25 mm (metallic mesh size 60, Metallurgical Industry Bertel, Caieiras, Brazil). Then, the microbiological analysis was performed using the method of the total count of mesophilic aerobes on plates and the method of the total count of molds and yeasts on plates [22] to evaluate the total heterotrophic count and mold and yeast count.

### 2.6. Statistical Analysis

Experimental data were analyzed by Statistica^®^ software (Stat Soft Inc., 10, Cary, NC, USA) and expressed as average values ± mean deviation. Significant differences among the means were evaluated by the Tukey test at *p* < 0.05.

## 3. Results

### 3.1. Physical, Chemical, Structural, and Barrier Characteristics of Membranes Subjected to Biodegradation

Table 1 exhibits the properties of membranes subjected to biodegradation (agar, chitosan and agar + chitosan). Physical, chemical, structural, and barrier characterization is extremely important for understanding the biodegradation specificity of membranes. The synthesized membranes did not show a significant difference (*p* < 0.05) in thickness (Table 2), which was already expected since the same proportion of solids was used in all formulations. The membranes used in the food sector generally have the same thickness as found in this study, approximately 0.07 mm [7,23,24]. Thickness is a critical property, as it is directly related to other membrane characteristics, such as tensile strength, water vapor permeability, and light transmittance [25].

Mechanical properties can be associated with the maximum tension applied to the membrane for its rupture and the maximum supported elongation; these are very important and variable properties depending on the application of interest [26]. The maximum tensile strength properties found in this study (4.99 to 10.84 MPa) are similar to those of films used in food applications. Oliveira et al. [27] obtained a rupture stress of 9.30 MPa in starch films extracted from mango seeds for use as packaging for various foods, and Martiny et al. [28] found 11.83 MPa in carrageenan films for preserving lamb meat. The maximum elongation (19.63 to 45.97%) is also similar to films used in research aimed at applications in the food industry. Rani et al. [29] obtained elongation at break from 1.87 to 35.52% in mustard, soybean bran, and linseed films.

Water vapor permeability is primarily evaluated in membranes for food applications, as it determines the barrier capacity to moisture, allowing food to either lose or absorb water vapor from the external environment [30,31]. However, in food products, low water vapor permeability is desirable to maintain the product’s sensory characteristics [32]. The membranes in this study showed low values for this parameter (Table 2). Li et al. [33] obtained a water vapor permeability of 1.31 × 10^−14^ (kg·m^−1^·Pa^−1^·s^−1^) in a biocomposite film of chitosan and fish skin gel for food applications.

Solubility is also an important parameter in the characterization of films for food packaging, as they must act as a protective layer against the external environment. Foods with high water activity require a highly water-resistant protective layer [34]. Furthermore, the biodegradability of biopolymeric membranes is also influenced by their solubility in water. In the degradation process, the compounds present in the membranes become accessible to the soil microbiota, facilitating biodegradation [35]. In this study, the solubility of the membrane-forming matrices ranged from 28.29% to 34.03%, considered as low solubility and suitable for application as food packaging, as reported by Li et al. [33], who developed blends of chitosan and fish skin gelatin with a solubility of 32.11%. Avila et al. [5] also developed chitosan membranes with zein fiber added with jaboticaba peel extract, exhibiting 27.38% solubility, for use in food applications.

**Figure 3:**
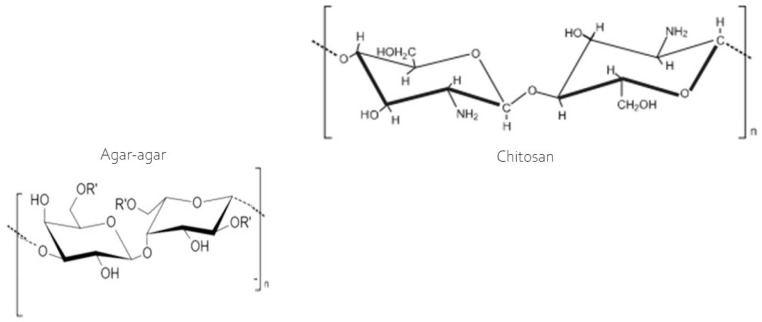
Molecular structure of the chitosan and agar [38].

The swelling index exhibited considerable variability among the developed membranes (Table 2), with the chitosan membrane absorbing the greatest amount of water, ranging from 320% to 440% between 2 and 60 min of analysis. This can be explained by the hydroxyl groups present in chitosan (Figure 3) as the sole film-forming polymer, which does not bind to the acetic acid used as a diluent, remaining ‘free’ to react with water. This explains the high swelling capacity of the membrane. However, in the blend, during the casting of the two polymers, according to Contessa et al. [26] the hydroxyl groups of chitosan participate in hydrogen bonds and electrostatic interactions between the polymers, making them unavailable for binding with water during the swelling analysis. However, the agar and the agar + chitosan blend varied from 47.62% to 73.87% and 57.12% to 106.74%, respectively, in the same analysis time frame. The swelling property indicates the membrane’s ability to retain water, and the smaller this capacity, the more effective the barrier will be between the food and the external environment. In this regard, the agar membrane is most suitable for application, followed by the blend (agar + chitosan). These results agree with those reported by Contessa et al. [7] for the agar + chitosan blend, who obtained around 67%, and [36] found approximately 400% for a gelatin membrane. The chitosan membrane, however, is not suitable for applications involving food products but can easily be applied in the case of dressings for healing skin. According to Li et al. [37] the bands observed are consistent with topical dressing applications, which require high swelling properties to efficiently absorb and eliminate wound exudates and accelerate healing.

The contact angle of the films indicates their affinity with water and the wettability of their surface. An angle greater than 90° denotes a more hydrophobic nature, with a lower affinity for water, while angles less than 90° characterize hydrophilic surfaces with high wettability [39,40]. It is observed that all three films have an affinity for water; the agar film, however, is less hydrophilic. The contact angle is in line with the values obtained for solubility, where agar also presented the lowest solubility, while chitosan presented the highest solubility value and the smallest angle, demonstrating an affinity with water. This observation is consistent with the degree of swelling, which was the highest among the membranes analyzed (Table 2).

Based on the discussed properties as well as those described in previous studies [7,41], the agar, chitosan, and agar + chitosan membranes meet the criteria of suitability for food packaging applications, particularly within industries that contribute significantly to solid waste generation. Following the COVID-19 outbreak, this contribution increased to an average of 85% of the packaging already being produced [42]. With a global focus on reducing the production of plastics, natural polymers are gradually replacing traditional petroleum-based plastics [43]. In this regard, agar, chitosan, and agar + chitosan membranes, such as those developed in this study, contribute to meeting the United Nations Sustainable Development Goals (SDGs). These goals aim to achieve sustainable management and efficient use of natural resources by 2030, ensuring responsible production and environmental conservation [44].

### 3.2. Structural Property of Membranes after Biodegradation in Soil

The soil used in the biodegradation analysis was obtained from an earthmoving industry and had an organic matter content of 12.36% (Table 3). Oberlintner et al. [45] found organic matter content of 15.8%, 25%, and 59.3% for industrial compost soil from waste management, vineyard soil, and gardening soil, respectively. The initial microbiota, represented by the presence of mesophilic bacteria, molds, and yeasts (Table 3), showed lower counts than those described by Filipini et al. [46], who obtained counts of 7 × 10^9^ CFU/g and 9 × 10^5^ CFU/g, respectively.

A decline in the count of mesophiles, molds, and yeasts in the natural soil was observed. This behavior may be linked to a possible decrease in nutrients available for consumption by microorganisms and for maintaining their life cycle, or even a reduction in aerobic conditions, as the experimental soil was not periodically agitated to maintain uniform conditions, as conducted by Oberlintner et al. [45]. However, the sterilized soil showed an initial increase in the count of the analyzed microbiota, a behavior possibly explained by the thermal treatment used to sterilize the soil, which degraded some macronutrients and consequently increased the availability of micronutrients necessary for the microorganisms’ life cycle [47]. The presence of viable microbiota in the sterilized soil is attributed to soil manipulation during the analysis period, as aseptic conditions were not maintained after the autoclaving process.

**Figure 4:**
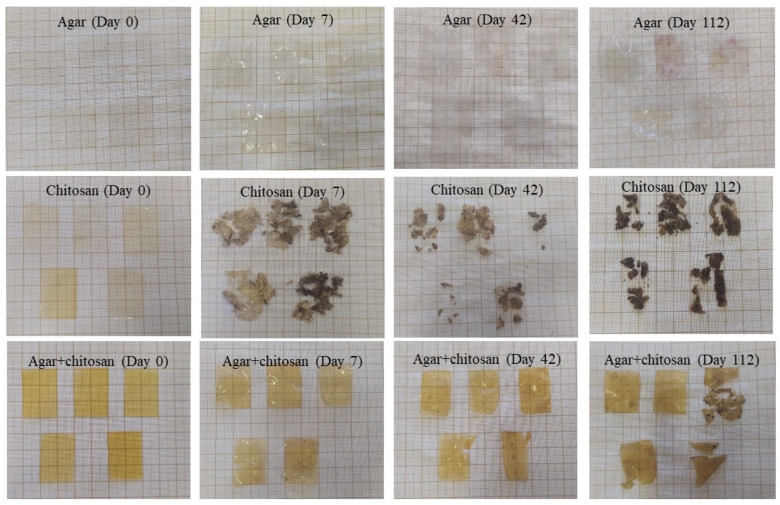
Visual appearance of agar, chitosan, and agar + chitosan membranes during biodegradation in sterilized soil.

**Figure 5:**
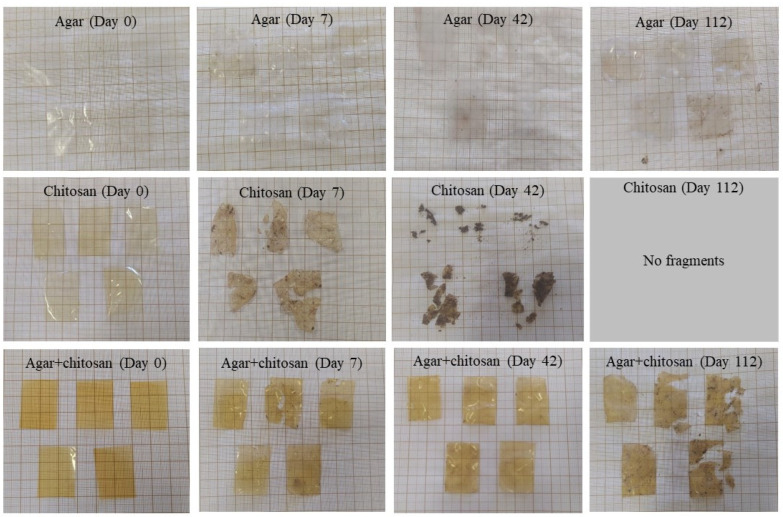
Visual appearance of agar, chitosan, and agar + chitosan membranes during biodegradation in natural soil.

The presence of microorganisms in the initially sterilized soil provided biodegradation conditions for the membranes (Figure 4). Up to 42 days, the thickness continuously decreased (Table 4) due to the increase in mesophilic bacteria (Table 3). After 42 days, the agar membranes began to interact with the soil, causing soil particles to adhere during measurement and resulting in an increase in thickness. The same behavior was observed in the agar + chitosan membrane; however, there was greater difficulty in separating the soil particles from the already highly degraded membrane (Figure 4). Furthermore, after 42 days, it became impossible to measure the thickness of the chitosan membrane, as it was significantly degraded. The same pattern was observed in the natural soil (Figure 5). The chitosan membrane exhibited faster degradation compared to the agar + chitosan membrane, followed by the agar membrane. This difference in degradation rates could be attributed to variations in water solubility and swelling properties among the membranes (Table 2). According to Byaruhanga et al. [48], the presence of moist soil may favor the breaking of hydrogen bonds and hydrophobic interactions, resulting in the breakdown of the molecule and greater availability for the present microbiota to act.

**Figure 6:**
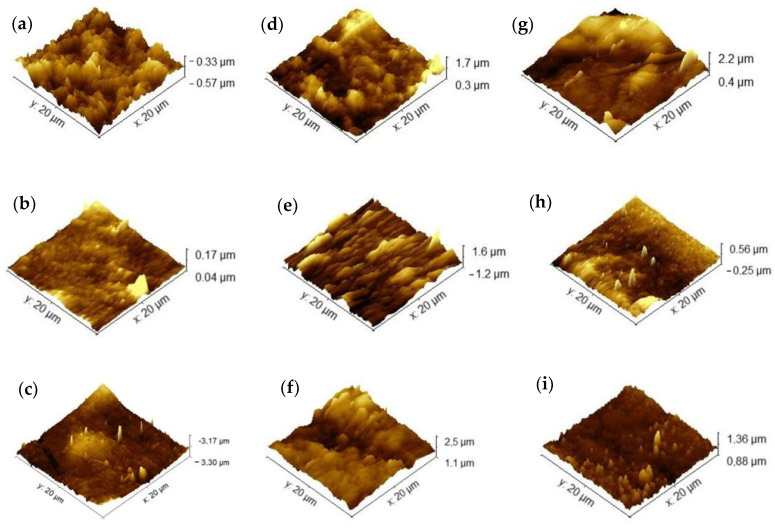
Atomic force microscopy of agar membranes: (**a**) agar, (**b**) chitosan, (**c**) agar + chitosan, before biodegradation, (**d**–**f**) after biodegradation in natural soil, and (**g**–**i**) after biodegradation in sterilized soil.

The surface morphology of the samples (Figure 6) represents the topography of the samples. The intensity of coloring indicates depth, with darker shades indicating greater distance between the sample and the equipment probe. An increase in regions with greater depth in the membranes after immersion in the soil is observed, also highlighted by the increase in roughness when compared to the control membranes without insertion in the soil. These morphological changes occur during the microbial degradation process. Tribedi and Sil [49] reported that soil bacteria are likely responsible for producing enzymes that create cavities in membranes, facilitating their breakdown and enabling bacteria to access and utilize the nutrients within the membranes without requiring prior oxidation. Similar behavior was observed by Samanta et al. [50] in polyethylene films, where an increase in the porosity of the membranes was observed after the biodegradation process in soil, which was related to the improvement in the wettability of the membranes during the process.

In this study, we can attribute the significant biodegradation observed in the chitosan membrane (Figure 4 and Figure 5) to its high affinity for water compared to the other membranes investigated. This affinity is supported by its lower contact angle and higher solubility in water (Table 2), as well as its increased roughness following biodegradation in natural soil.

**Figure 7:**
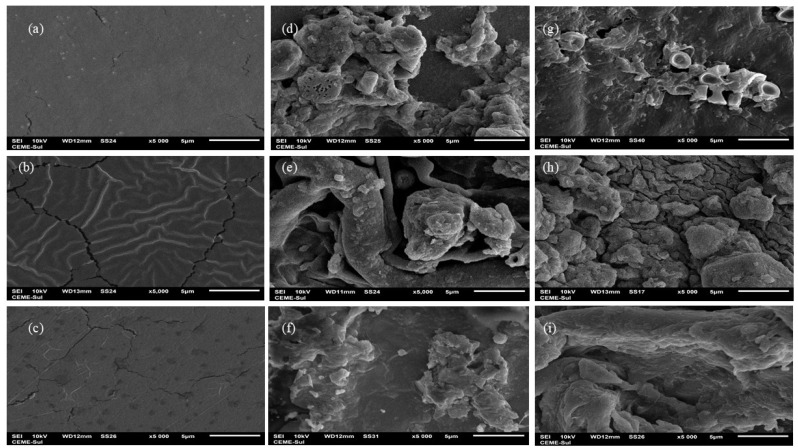
Scanning electron microscopy: (**a**–**c**) agar, chitosan, and agar + chitosan membranes without the biodegradation process, (**d**–**f**) agar, chitosan, and agar + chitosan membranes after the biodegradation process in natural soil, and (**g**–**i**) agar, chitosan, and agar + chitosan membranes after the biodegradation process in sterilized soil; all images at 5000× magnification.

In addition to surface morphology analysis, scanning electron microscopy (SEM) imaging (Figure 7) highlighted notable differences between the membranes before and after biodegradation in soil. Visually, the agar membrane did not show clues of biodegradation. However, SEM images illustrated a distinct alteration in the surface characteristics: the initially homogeneous and compact surface with minor fissures (Figure 7a) presented visible transformation following the biodegradation process (Figure 7d,g). The images revealed the presence of the membrane alongside a mixture of soil particles, and potentially fungal and yeast elements, indicating the progression of degradation and microbial activity on the membrane surface. Similar phenomena were observed by Ali et al. [51] and Khruengsai et al. [52] during the degradation of low-density polyethylene films. Generally, all membranes displayed a significant structural change, likely due to enzymatic depolymerization facilitated by the microbiota, resulting in increased roughness in the membranes [50], as observed in the morphological analysis, and loss of mass, which was evident from day 42 onwards in the chitosan and agar + chitosan membranes, as depicted in Figure 3 and Figure 4. The agar membrane likely initiated biodegradation at the time of analysis, as indicated by energy-dispersive X-ray spectroscopy (EDS) analysis, which revealed the presence of elements such as Al, Pb, F, Na, Mg, Al, Si, and K (Supplementary Materials Table S1) in the membranes subjected to biodegradation but not observed in the control membrane.

**Figure 8:**
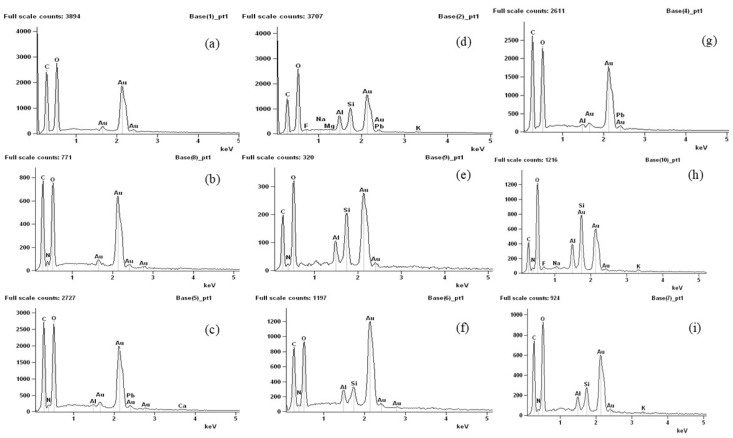
Energy dispersive spectroscopy: (**a**–**c**) agar, chitosan, and agar + chitosan membranes without the biodegradation process, (**d**–**f**) agar, chitosan, and agar + chitosan membranes after the biodegradation process in natural soil, and (**g**–**i**) agar, chitosan, and agar + chitosan membranes after the biodegradation process in sterilized soil.

By means of energy dispersive spectroscopy analysis (Figure 8), it was possible to detect the presence of elements in the biodegraded samples that were not present in the control samples. Manganese (Mg), aluminum (Al), silicon (Si), calcium (Ca), phosphorus (P), and sodium (Na) were detected and may be linked to the presence of soil particles, as these micronutrients are commonly found in soils of the region [53,54,55]. The presence of carbon in the control samples, originating from the components of the film-forming matrix, showed a reduction after the membranes were inserted into the soil, suggesting that it was consumed during the biodegradation process. The presence of soil particles in the analyzed samples was associated with the biodegradation process, as the partially degraded samples analyzed via SEM and TEM exhibited a mixture of membrane and soil particles.

Based on our findings, it was possible to observe that the chitosan membrane showed biodegradation within the shortest period, becoming evident after 7 days of contact with the soil, followed by the agar + chitosan membrane, where a slight change could be visually noticed after 7 days. No visual changes were observed in the agar membrane; however, atomic force microscopy and scanning electron microscopy analyses revealed structural alterations, including indications of soil particles, as confirmed by energy dispersive spectroscopy analysis.

The shorter initiation times of the biodegradation process were associated with higher water solubility and swelling values (Table 2). Generally, the biodegradability of biopolymeric membranes may be linked to their affinity with water and can, or not, achieve complete biodegradation within 180 days, as determined by regulations such as ASTM D6400 [56] and ASTM D6868 [57]. However, when compared to low-density polyethylene films, which degrade at a rate of 0.2% by weight every 10 years [45,58], biopolymeric membranes offer the advantage of minimal impact on the terrestrial environment.

## 4. Conclusions

Strategies for developing new materials for food packaging to reduce the environmental impact generated by synthetic packaging are highly exploited, with an emphasis on biopolymeric packaging. In this study, it was possible to evaluate the physical, mechanical, and barrier properties of three different biopolymeric membranes, chitosan, agar-agar, and the chitosan/agar-agar blend.

Regarding mechanical properties, the agar-agar membrane and the agar-agar/chitosan blend presented higher values than the chitosan membrane, without significant differences between them. However, the chitosan membrane presented the best value of elongation at break. Besides that, the agar-agar membrane showed great water resistance, with better results than the others, presenting lower solubility in water and swelling. Water vapor permeability and the contact angle were lower for the chitosan membrane than the others. It was possible to observe that the addition of chitosan to the agar-agar/chitosan blend promoted a reduction in water vapor permeability, while the contact angle did not show significant differences between samples.

It was verified that the materials developed showed promising characteristics for application as food packaging material. Although they are biopolymeric materials, a biodegradation study must be considered, mainly evaluating the influence of composition on this process. In this sense, the chitosan membrane showed a greater tendency to biodegradability in soil (natural and sterilized) followed by the blend and the agar-agar membrane, respectively. These results are supported by scanning electron microscopy, atomic force microscopy, and energy dispersive spectroscopy analyses, which demonstrated changes in the membranes surfaces.

Therefore, based on biodegradability analyses, it is possible to state that the biopolymeric materials developed in the present study present promising properties for application as food packaging. Furthermore, it was proven that the materials meet the requirements for biodegradable material, as they showed biodegradability in less than 180 days.

## Figures and Tables

**Table 1:** Compositions of biopolymeric membranes.

Membrane	Chitosan (g)	Agar-Agar (g)	Glycerol (g)
**Agar-agar**	-	1	0.3
**Chitosan**	1	-	0.3
**Blend**	0.5	0.5	0.3

**Table 2:** Physical, mechanical, and barrier characterization of biopolymeric membranes.

	Biopolymeric Matrix		
	Agar	Chitosan	Blend (Agar + Chitosan)
Thickness (mm)	0.07 ± 0.01 ^a^	0.07 ± 0.01 ^a^	0.07 ± 0.00 ^a^
Maximum Breaking Stress (MPa)	10.84 ± 0.81 ^a^	4.99 ± 0.39 ^b^	9.61 ± 0.57 ^a^
Elongation (%)	24.44 ± 4.55 ^b^	45.97 ± 5.76 ^a^	19.63 ± 2.26 ^b^
Water vapor permeability (kg·m^−1^·s^−1^·Pa^−1^)	2.18 × 10^−12^ ± 2.26 × 10^−15 a^	6.07 × 10^−14^ ± 4.57 × 10^−15 b^	5.23 × 10^−14^ ± 7.89 × 10^−16 c^
Water solubility (%)	28.29 ± 0.55 ^c^	34.03 ± 0.90 ^a^	30.93 ± 0.86 ^b^
Swelling (%)			
2 (min)	57.12 ± 2.08 ^b^	320.84 ± 8.87 ^a^	47.62 ± 2.89 ^c^
5 (min)	60.18 ± 0.95 ^b^	371.16 ± 7.62 ^a^	51.04 ± 1.94 ^b^
30 (min)	68.84 ± 1.09 ^b^	381.43 ± 11.71 ^a^	52.25 ± 0.16 ^b^
60 (min)	73.87 ± 1.39 ^c^	440.32 ± 2.76 ^a^	106.74 ± 18.97 ^b^
Contact Angle	43.46 ± 6.74 ^a^	36.07 ± 2.56 ^a^	38.56 ± 6.58 ^a^

Values are presented as mean ± standard deviation. Different letters on the same line indicate a significant difference (*p* < 0.05).

**Table 3:** Characterization of soil and sterilized soil.

Soil	
**Organic Matter Content (%)**	12.36 ± 0.53
Moisture Content (%)	25.03 ± 1.01
Mesophilic aerobes (CFU/mL)	
7 Days	1.57 × 10^6^
45 Days	4.09 × 10^5^
115 Days	7.70 × 10^3^
Molds and yeasts (CFU/g)	
7 Days	9.60 × 10^4^
45 Days	1.29 × 10^3^
115 Days	5.20 × 10^3^
Sterilized soil	
Mesophilic aerobes (CFU/mL)	
7 Days	1.00 × 10^3^
45 Days	3.60 × 10^6^
115 Days	8.60 × 10^4^
Molds and yeasts (CFU/g)	
7 Days	3.00 × 10^4^
45 Days	2.86 × 10^5^
115 Days	7.00 × 10^3^

**Table 4:** Membrane thickness before and after the biodegradation process.

Days	Natural Soil (mm)	Sterile Soil (mm)
Agar membrane (control) = 0.09 ± 0.01 mm		
7	0.09 ± 0.01	0.07 ± 0.01
14	0.08 ± 0.02	0.08 ± 0.03
21	0.04 ± 0.01	0.08 ± 0.02
28	0.04 ± 0.01	0.07 ± 0.01
42	0.04 ± 0.02	0.06 ± 0.02
70	0.03 ± 0.01	0.07 ± 0.01
112	0.03 ± 0.00	0.07 ± 0.00
Chitosan membrane (control) = 0.08 ± 0.01 mm		
7	0.07 ± 0.01	0.08 ± 0.01
14	0.14 ± 0.01	0.13 ± 0.02
21	0.13 ± 0.02	0.13 ± 0.02
28	0.12 ± 0.02	0.13 ± 0.01
42	-	-
70	-	-
112	-	-
Agar + Chitosan membrane (control) = 0.075 ± 0.06 mm		
7	0.06 ± 0.01	0.06 ± 0.01
14	0.07 ± 0.01	0.05 ± 0.01
21	0.05 ± 0.01	0.07 ± 0.01
28	0.05 ± 0.01	0.06 ± 0.01
42	0.04 ± 0.01	0.03 ± 0.01
70	0.06 ± 0.01	0.06 ± 0.01
112	0.05 ± 0.01	0.06 ± 0.01

## Data Availability

Data are contained within the article and supplementary materials.

## Supplementary Materials

The following supporting information can be downloaded at: https://www.mdpi.com/article/10.3390/polym16202940/s1, Table S1: Elemental analysis of membranes before and after the biodegradation process in soil, by means of energy dispersive spectroscopy.

## References

[1] Brooks A.L., Wang S., Jambeck J.R. (2018). The Chinese Import Ban and Its Impact on Global Plastic Waste Trade.

[2] Geyer R., Jambeck J.R., Law K.L. (2017). Production, Use, and Fate of All Plastics Ever Made.

[3] Masmoudi F., Bessadok A., Dammak M., Jaziri M., Ammar E. (2016). Biodegradable Packaging Materials Conception Based on Starch and Polylactic Acid (PLA) Reinforced with Cellulose. Environ. Sci. Pollut. Res..

[4] Walker T.R., Fequet L. (2023). Current Trends of Unsustainable Plastic Production and Micro(Nano)Plastic Pollution. TrAC Trends Anal. Chem..

[5] Avila L.B., Pinto D., Silva L.F.O., de Farias B.S., Moraes C.C., Da Rosa G.S., Dotto G.L. (2022). Antimicrobial Bilayer Film Based on Chitosan/Electrospun Zein Fiber Loaded with Jaboticaba Peel Extract for Food Packaging Applications. Polymers.

[6] Avila L.B., Barreto E.R.C., Moraes C.C., Morais M.M., da Rosa G.S. (2022). Promising New Material for Food Packaging: An Active and Intelligent Carrageenan Film with Natural Jaboticaba Additive. Foods.

[7] Contessa C.R., da Rosa G.S., Moraes C.C. (2021). New Active Packaging Based on Biopolymeric Mixture Added with Bacteriocin as Active Compound. Int. J. Mol. Sci..

[8] Madni A., Kousar R., Naeem N., Wahid F. (2021). Recent Advancements in Applications of Chitosan-Based Biomaterials for Skin Tissue Engineering. J. Bioresour. Bioprod..

[9] Naveed M., Phil L., Sohail M., Hasnat M., Baig M.M.F.A., Ihsan A.U., Shumzaid M., Kakar M.U., Mehmood Khan T., Akabar M.D. (2019). Chitosan Oligosaccharide (COS): An Overview. Int. J. Biol. Macromol..

[10] Pervez S., Nawaz M.A., Jamal M., Jan T., Maqbool F., Shah I., Aman A., Ul Qader S.A. (2019). Improvement of Catalytic Properties of Starch Hydrolyzing Fungal Amyloglucosidase: Utilization of Agar-Agar as an Organic Matrix for Immobilization. Carbohydr. Res..

[11] Shankar S., Reddy J.P., Rhim J.W. (2015). Effect of Lignin on Water Vapor Barrier, Mechanical, and Structural Properties of Agar/Lignin Composite Films. Int. J. Biol. Macromol..

[12] Nair N.R., Sekhar V.C., Nampoothiri K.M., Pandey A. (2016). Biodegradation of Biopolymers. Current Developments in Biotechnology and Bioengineering: Production, Isolation and Purification of Industrial Products.

[13] Swetha T.A., Bora A., Mohanrasu K., Balaji P., Raja R., Ponnuchamy K., Muthusamy G., Arun A. (2023). A Comprehensive Review on Polylactic Acid (PLA) Synthesis, Processing and Application in Food Packaging. Int. J. Biol. Macromol..

[14] Amariei S., Ursachi F., Petraru A. (2022). Development of New Biodegradable Agar-Alginate Membranes for Food Packaging. Membranes.

[15] Fonseca-García A., Jiménez-Regalado E.J., Aguirre-Loredo R.Y. (2021). Preparation of a Novel Biodegradable Packaging Film Based on Corn Starch-Chitosan and Poloxamers. Carbohydr. Polym..

[16] Soares J.M.A., Silva Júnior E.D., de Veras B.O., Yara R., de Albuquerque P.B.S., de Souza M.P. (2021). Active Biodegradable Film Based on Chitosan and Cenostigma Nordestinum ’ Extracts for Use in the Food Industry. J. Polym. Environ..

[17] (2012). Standard Test Method for Tensile Properties of Thin Plastic Sheeting.

[18] Gontard N., Guilbert S., Cuq J.-L. (1992). Edible Wheat Gluten Films: Influence of the Main Process Variables on Film Properties Using Response Surface Methodology. J. Food Sci..

[19] Bunhak É.J., Mendes E.S., Pereira N.C., Cavalcanti O.A. (2007). Influência Do Sulfato de Condroitina Na Formação de Filmes Isolados de Polimetacrilato: Avaliação Do Índice de Intumescimento e Permeabilidade Ao Vapor d’água. Quim. Nova.

[20] (2016). Standard Test Methods for Water Vapor Transmission of Materials 1.

[21] Martucci J.F., Ruseckaite R.A. (2010). Biodegradable Three-Layer Film Derived from Bovine Gelatin. J. Food Eng..

[22] da Silva N., Junqueira V.C.A., de Arruda Silveira N.F., Taniwaki M.H., Gomes R.A.R., Okazaki M.M. (2018). Manual de Métodos de Análise Microbiológica de Alimentos e Água.

[23] Kaya M., Ravikumar P., Ilk S., Mujtaba M., Akyuz L., Labidi J., Salaberria A.M., Cakmak Y.S., Erkul S.K. (2018). Production and Characterization of Chitosan Based Edible Films from Berberis Crataegina’s Fruit Extract and Seed Oil. Innov. Food Sci. Emerg. Technol..

[24] Roy S., Rhim J.W. (2021). Preparation of Gelatin/Carrageenan-Based Color-Indicator Film Integrated with Shikonin and Propolis for Smart Food Packaging Applications. ACS Appl. Biol. Mater..

[25] Wang X., Yong H., Gao L., Li L., Jin M., Liu J. (2019). Preparation and Characterization of Antioxidant and PH-Sensitive Films Based on Chitosan and Black Soybean Seed Coat Extract. Food Hydrocoll..

[26] Contessa C.R., Rosa G.S.d., Moraes C.C., Burkert J.F. (2023). de M. Agar-Agar and Chitosan as Precursors in the Synthesis of Functional Film for Foods: A Review. Macromol.

[27] Oliveira D., Rosa D., Zavareze G.S. (2018). Terms and Conditions Privacy Policy Search: (TITLE-ABS-KEY(Agroindustria)) AND (Agroindustria) 1) Deamici Development of Cookies from Agroindustrial by-Products [Article@Desenvolvimento de Biscoitos a Partir de Subprodutos Da Agroindústria]. Rev. Bras. Frutic..

[28] Martiny T.R., Raghavan V., de Moraes C.C., da Rosa G.S., Dotto G.L. (2020). Bio-Based Active Packaging: Carrageenan Film with Olive Leaf Extract for Lamb Meat Preservation. Foods.

[29] Rani R., Gosh T., Badwaik L.S. (2023). Optimization of Mustard, Soybean and Flaxseed Meal Blend Formulation for Development of Biopolymeric Film and Its Characterization. Sustain. Chem. Pharm..

[30] Long J., Zhang W., Zhao M., Ruan C.Q. (2023). The Reduce of Water Vapor Permeability of Polysaccharide-Based Films in Food Packaging: A Comprehensive Review. Carbohydr. Polym..

[31] Alavi T., Rezvanian M., Ahmad N., Mohamad N., Ng S.F. (2019). Pluronic-F127 Composite Film Loaded with Erythromycin for Wound Application: Formulation, Physicomechanical and in Vitro Evaluations. Drug Deliv. Transl. Res..

[32] Tavassoli M., Khezerlou A., Punia Bangar S., Bakhshizadeh M., Haghi P.B., Moghaddam T.N., Ehsani A. (2023). Functionality Developments of Pickering Emulsion in Food Packaging: Principles, Applications, and Future Perspectives. Trends Food Sci. Technol..

[33] Li Y., Tang C., He Q. (2021). Effect of Orange (*Citrus sinensis* L.) Peel Essential Oil on Characteristics of Blend Films Based on Chitosan and Fish Skin Gelatin. Food Biosci..

[34] Huang X., Luo X., Liu L., Dong K., Yang R., Lin C., Song H., Li S., Huang Q. (2020). Formation Mechanism of Egg White Protein/κ-Carrageenan Composite Film and Its Application to Oil Packaging. Food Hydrocoll.

[35] Carissimi M., Flôres S.H., Rech R. (2018). Effect of Microalgae Addition on Active Biodegradable Starch Film. Algal Res..

[36] Wang L.F., Rhim J.W. (2015). Preparation and Application of Agar/Alginate/Collagen Ternary Blend Functional Food Packaging Films. Int. J. Biol. Macromol..

[37] Li D., Ye Y., Li D., Li X., Mu C. (2016). Biological Properties of Dialdehyde Carboxymethyl Cellulose Crosslinked Gelatin-PEG Composite Hydrogel Fibers for Wound Dressings. Carbohydr. Polym..

[38] Diop C.I.K., Beltran S., Sanz M.T., Garcia-Tojal J., Trigo-lopez M. (2023). Designing Bilayered Composite Films by Direct Agar/Chitosan and Citric Acid-Crosslinked PVA/Agar Layer-by-Layer Casting for Packaging Applications. Food Hydrocoll.

[39] Kanmani P., Rhim J.W. (2014). Antimicrobial and Physical-Mechanical Properties of Agar-Based Films Incorporated with Grapefruit Seed Extract. Carbohydr. Polym..

[40] Yuan Y., Lee T.R. (2013). Contact Angle and Wetting Properties. Springer Ser. Surf. Sci..

[41] Contessa C.R., de Souza N.B., Gonçalo G.B., de Moura C.M., da Rosa G.S., Moraes C.C. (2021). Development of Active Packaging Based on Agar-Agar Incorporated with Bacteriocin of *Lactobacillus sakei*. Biomolecules.

[42] Jang Y., Nam Kim K., Woo J.R. (2023). Post-Consumer Plastic Packaging Waste from Online Food Delivery Services in South Korea. Waste Manag..

[43] Quan C., Chen C., Gao N., Liu D. (2024). Enhancing the Volatile Fatty Acids Production from Food Waste as Well as Polylactate Plastic Degradation by Coupled with Hydrothermal Pretreatment. Fuel.

[44] Hassoun A., Boukid F., Ozogul F., Aït-Kaddour A., Soriano J.M., Lorenzo J.M., Perestrelo R., Galanakis C.M., Bono G., Bouyahya A. (2023). Creating New Opportunities for Sustainable Food Packaging through Dimensions of Industry 4.0: New Insights into the Food Waste Perspective. Trends Food Sci. Technol..

[45] Oberlintner A., Bajić M., Kalčíková G., Likozar B., Novak U. (2021). Biodegradability Study of Active Chitosan Biopolymer Films Enriched with Quercus Polyphenol Extract in Different Soil Types. Environ. Technol. Innov..

[46] Filipini S., Romani V.P. (2020). Food Hydrocolloids Biodegradable and Active-Intelligent Films Based on Methylcellulose and ~ o (*Syzygium cumini*) Skins Extract for Food Packaging. Food Hydrocoll..

[47] Xu E., Wang J., Tang J., Ruan S., Ma S., Qin Y., Wang W., Tian J., Zhou J., Cheng H. (2021). Heat-Induced Conversion of Multiscale Molecular Structure of Natural Food Nutrients: A Review. Food Chem..

[48] Byaruhanga Y.B., Erasmus C., Taylor J.R.N. (2005). Effect of Microwave Heating of Kafirin on the Functional Properties of Kafirin Films. Cereal Chem..

[49] Tribedi P., Sil A.K. (2013). Low-Density Polyethylene Degradation by Pseudomonas Sp. AKS2 Biofilm. Environ. Sci. Pollut. Res..

[50] Samanta S., Datta D., Halder G. (2020). Biodegradation Efficacy of Soil Inherent Novel sp. Bacillus Tropicus (MK318648) onto Low Density Polyethylene Matrix. J. Polym. Res..

[51] Ali S.A., Zakarya S., Khaled S. (2022). Screening and Optimisation of the Biodegradation Potential for Low Density Polyethylene (LDPE) Films by Fusarium Equiseti and Brevibacillus Parabrevis. Biosci. Biotechnol. Res. Asia.

[52] Khruengsai S., Sripahco T., Pripdeevech P. (2021). Low-Density Polyethylene Film Biodegradation Potential by Fungal Species from Thailand. J. Fungi.

[53] Coedert’ W.J., Beatty’ M.T. (1971). Caracterjzação de Grumussolos no Sudoeste do rio Grande do sul. ii. Mineralogia e Gênese. Pesqui. Agropecuária Bras..

[54] Souza M.R.d., Hilário Garcia A.L., Dalberto D., Martins G., Picinini J., Souza G.M.S.d., Chytry P., Dias J.F., Bobermin L.D., Quincozes-Santos A. (2021). Environmental Exposure to Mineral Coal and By-Products: Influence on Human Health and Genomic Instability. Environ. Pollut..

[55] Flores C.G., Schneider H., Marcilio N.R., Ferret L., Oliveira J.C.P. (2017). Potassic Zeolites from Brazilian Coal Ash for Use as a Fertilizer in Agriculture. Waste Manag..

[56] (2022). American Society for Testing and Materials. *Standard Specification for Labeling of Plastics Designed to Be Aerobically Composted in Municipal or Industrial Facilities 1*.

[57] (2011). Standard Specification for Labeling of End Items That Incorporate Plastics and Polymers as Coatings or Additives with Paper and Other Substrates Designed to Be Aerobically Composted in Municipal or Industrial Facilities 1.

[58] Albertsson A.C. (1980). The Shape of the Biodegradation Curve for Low and High Density Polyethenes in Prolonged Series of Experiments. Eur. Polym. J..

