# Supplementary material for: Biodegradation Study of Food Packaging Materials: Assessment of the Impact of the Use of Different Biopolymers and Soil Characteristics
Source: Polymers (Basel). 2024 Oct 20;16(20):2940. doi: 10.3390/polym16202940 (PMC11511331; doi:10.3390/polym16202940)
Supplement: Supplementary file 1 [file polymers-16-02940-s001.zip › polymers-3242906-supplementary.pdf]

Table S1 - Elemental analysis of membranes before and after the biodegradation process in soil, by energy dispersive spectroscopy

Membrane	Element	Mass (%)
Agar (control)	C<br>O<br>Au	13,01<br>29,75<br>57,25
Agar (biodegradation in natural soil)	C<br>O<br>F<br>Na<br>Mg<br>Al<br>Si<br>K<br>Au<br>Pb	6,71<br>25,46<br>0,05<br>0,06<br>0,085<br>5,61<br>11,28<br>2,51<br>46,41<br>0,81
Agar (biodegradation in sterilized soil)	C<br>O<br>Al<br>Au<br>Pb	17,28<br>23,89<br>0,50<br>56,79<br>1,52
Chitosan (control)	C<br>N<br>O<br>Au	12,01<br>4,25<br>26,61<br>57,13
Chitosan (biodegradation in natural soil)	C<br>N<br>O<br>Al<br>Si<br>Au	6,22<br>1,60<br>21,71<br>4,24<br>13,19<br>53,03
Chitosan (biodegradation in sterilized soil)	C<br>N<br>O<br>F<br>Na<br>Mg<br>Al<br>Si<br>K<br>Au	4,88<br>1,88<br>28,68<br>0,52<br>0,43<br>0,73<br>5,25<br>14,90<br>1,10<br>41,61
Agar+chitosan (control)	C<br>N<br>O<br>Al<br>Ca<br>Au	12,37<br>3,21<br>27,27<br>0,24<br>0,55<br>53,24

	Pb	3,12
Agar+chitosan (biodegradation in natural soil)	C	11,04
	N	1,97
	O	19,65
	Al	2,48
	Si	3,93
	K	0,38
	Ca	0,63
	Au	60,15
Agar+chitosan (biodegradation in sterilized soil)	C	10,29
	N	2,70
	O	28,7
	Al	3,03
	Si	11,60
	K	0,29
	Au	43,37
